# An Automatic Method to Reduce Baseline Wander and Motion Artifacts on Ambulatory Electrocardiogram Signals

**DOI:** 10.3390/s21248169

**Published:** 2021-12-07

**Authors:** Hongzu Li, Pierre Boulanger

**Affiliations:** Faculty of Science—Computing Science, University of Alberta, Edmonton, AB T6G 2R3, Canada; pierreb@ualberta.ca

**Keywords:** ambulatory electrocardiogram, signal processing, empirical mode decomposition, adaptive filter, noise removal

## Abstract

Today’s wearable medical devices are becoming popular because of their price and ease of use. Most wearable medical devices allow users to continuously collect and check their health data, such as electrocardiograms (ECG). Therefore, many of these devices have been used to monitor patients with potential heart pathology as they perform their daily activities. However, one major challenge of collecting heart data using mobile ECG is baseline wander and motion artifacts created by the patient’s daily activities, resulting in false diagnoses. This paper proposes a new algorithm that automatically removes the baseline wander and suppresses most motion artifacts in mobile ECG recordings. This algorithm clearly shows a significant improvement compared to the conventional noise removal method. Two signal quality metrics are used to compare a reference ECG with its noisy version: correlation coefficients and mean squared error. For both metrics, the experimental results demonstrate that the noisy signal filtered by our algorithm is improved by a factor of ten.

## 1. Introduction

Electrocardiogram (ECG) is one of the most used medical tools for cardiologists to detect heart anomalies. It is easy to use, painless for patients, and can collect heart information from many angles so that the cardiologists can make more accurate diagnoses. There are many types of electrocardiogram inputs such as one-lead, three-lead, and twelve-lead, etc. Among these lead systems, one-lead and three-lead ECG devices are often used for continuous heart monitoring. Today’s rapid development of cloud technology allows new medical products such as QardioMD [1], Vivalink [2], and Astroskin Smart Shirt [3] to measure numerous physiological indicators simultaneously and transmit this information to a remote database in the so-called cloud. Using this mobile technology, one can evaluate a patient’s condition as they perform their daily activities. It has been shown [4] that early detection of heart anomalies is critical to reducing the effects of severe heart conditions such as infarcts, angina, arterial fibrillation, and sudden cardiac arrest. Most mobile commercial healthcare devices can measure 1-lead or 3-lead ECG in addition to respiration rate, blood oxygenation, some approximation of blood pressure, and much more. Many mobile devices can continuously measure and transfer to the cloud patient’s health data for 24 to 48 h. Even though 1-lead and 3-lead ECGs contain less information than the standard 12-lead ECG, 24-h continuous monitoring is sufficient to detect heart anomalies [5].

**Figure 1:**
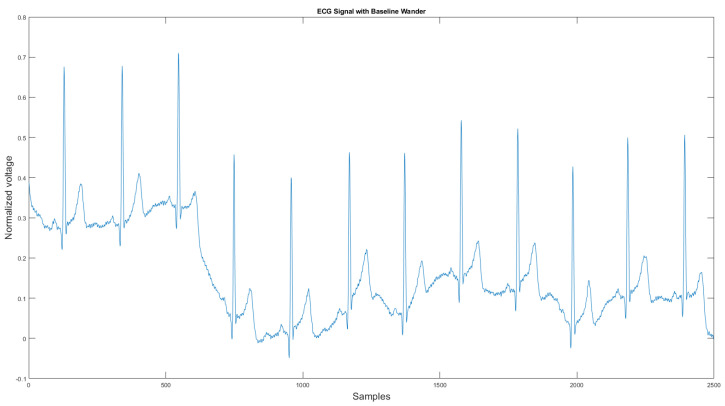
Baseline wander affected ECG signal.

**Figure 2:**
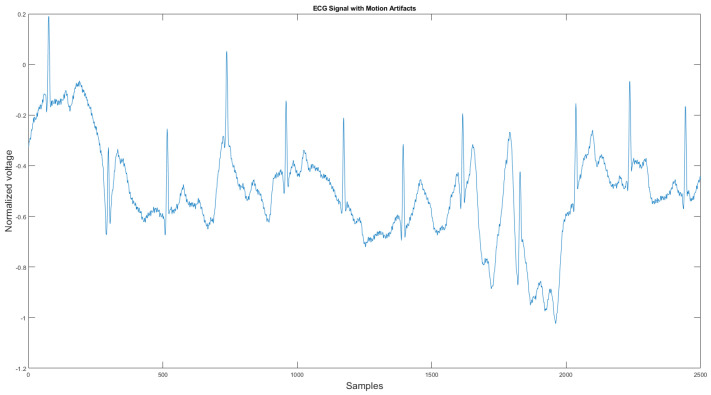
Motion artifact affected ECG signal.

The commercial personal medical device possesses many advantages such as ease of use, long-term body monitoring, and easy access to personal health data. However, it also has some important limitations. One of the major drawbacks is that the collected ECG is frequently contaminated with noise, making the ECG interpretation impossible. The standard 12-lead ECG device in clinics requires measuring the patient at rest, but most mobile healthcare devices use a rubber band to press the sensor onto the patient’s chest; therefore, the sensor is more likely to be affected by body motion. Body motion introduces two major noise types to the ECG signal: baseline wander (Figure 1) and motion artifact (Figure 2).

As shown in Figure 1 and Figure 2, ECG signals are distorted by artifacts that have nothing to do with the heart functions. In some cases, artifacts could even mimic true arrhythmia, leading to false diagnostics [6]. Therefore, noise removal is a necessary step for any ambulatory ECG monitoring applications to be useful. This paper proposes a new noise removal method that automatically suppresses the baseline wander and motion artifact. The new algorithm consists of an adaptive empirical mode decomposition and reconstruction algorithm capable of removing baseline wander and some motion artifacts, followed by an adaptive filter algorithm that uses an accelerometer to further reduce the motion artifacts. In Section 2.1, we will review the current state-of-the-art of noise removal techniques for ambulatory ECG. Section 2.2 describes the proposed algorithm. Then, in Section 3, the experimental testing setup and the results are presented. We then conclude in Section 4 by reviewing the advantages of our approach.

## 2. Materials and Methods

### 2.1. Related Work

There are four conventional methods for removing baseline wander and motion artifacts [7] that one can find in the literature. In the following sub-sections, we explain each technique and its implementations. Moreover, we will discuss the advantage and disadvantages of each method.

#### 2.1.1. Digital Filters

Digital filters are widely used to remove the unwanted frequency components in signals. The combination of different digital filters is called a filter bank. It combines various digital filters such as low-pass filter, high-pass filter, band-pass filter, notch filters, differentiator, and normalizer, etc. The ECG signal frequency component without noise range from 0.1 to 100 Hz [8]. To extract the pure ECG signal information, low bound and high bound filters must be applied according to the based ECG signal’s frequency range. One of the filter bank advantages is that it is straightforward to implement and is highly efficient. However, its drawback is that fixed digital filters could introduce nonlinear phase distortion and key points displacement, which could cause deformation on important waves of the ECG signal such as QRS complex [9]. Moreover, the parameters of the digital filter are tuned specifically for one dataset. It may not work well for the data collected from other devices. Furthermore, the digital filter does not perform well in removing the noises that have a similar frequency to the ECG signal.

#### 2.1.2. Discrete Wavelet Transform

Discrete wavelet transform (DWT) was first introduced by Mallat in 1989 [10]. It can be used to analyze non-stationary signals, such as ECG [11]. In [12], Poungponsri combines the DWT with an artificial neural network to remove a wide range of noise. DWT decomposes the signal into detail coefficients to approximate the signal using a wavelet mother function. Each coefficient corresponds to a different frequency range. Therefore, by observing the extracted approximation and detail coefficients, one can easily distinguish the coefficients that contain the ECG signal and the coefficients that contain the signal noise. One could only apply noise removal methods such as a digital filter to the noisy coefficients as the coefficients with the ECG signal will not be affected. Despite the great advantages of DWT, some drawbacks make it hard to use in practice. The first DWT requires to set and select the number of coefficients manually. The second is how to find the most suitable wavelet mother function, which could be different for different ECG signals. These limitations make it hard to automate such an algorithm.

#### 2.1.3. Empirical Mode Decomposition

Empirical mode decomposition (EMD) is an adaptive and entirely data-driven technique that obtains the oscillatory modes present in the data [13]. It was first introduced by Huang in 1998. Similar to DWT, EMD decomposes the ECG signal into many sub-band signals. The high-frequency components of the EMD decomposition are called intrinsic mode functions (IMF), and the low-frequency components are called residual. Unlike DWT, which relies on the mother wavelet functions, EMD does not need to find the best wavelet function or set the number of IMFs since this method is fully data-driven. In 2006, Weng applied the EMD method to the ECG signal to remove high-frequency noise with minimum signal distortion [14]. In 2008, Blanco proved the EMD method could be used for baseline wander correction [15]. However, the number of IMFs may differ for different ECG signals, implying that the IMFs do not have a fixed frequency range. Therefore, human intervention is often required to separate clean and noisy IMFs for signal reconstruction.

#### 2.1.4. Variational Mode Decomposition

The variational mode decomposition (VMD) was proposed by Dragoniretskiy and Zosso in 2013 [16]. It was an alternative approach to the empirical mode decomposition. Unlike the EMD method, the VMD method decomposes the signal from low frequency to high-frequency, where the residual contains the highest frequency component. Therefore, when dealing with the high-frequency noise in the signal, the VMD method performs better than the EMD method [17]. In 2016, Mohan applied the VMD method for the powerline interference on the ECG signal. However, since the VMD method is a variant of the EMD algorithm, human intervention is also required for clean IMF selection.

#### 2.1.5. Adaptive Filter

Adaptive filtering (AF) takes the original noisy signal and a reference noise signal as input. It then automatically adjusts the filter weights based on the reference noise signal to improve the signal reconstruction. There are three types of AF: least mean square (LMS) filter, normalized least mean square filter (NLMS), and recursive least squares filter (RLS). In 1991, Thakor et al. [18] first introduced the LMS adaptive filter to remove the baseline wander, 60 Hz power line noise, muscle noise, and motion wander. One shortcoming of the LMS adaptive filter is that it is sensitive to input scaling. Therefore, a power normalized least mean squares adaptive filter (NLMS) has been proposed to solve this problem by [19]. The last adaptive filter type is the recursive least square (RLS) adaptive filter. The RLS algorithm has excellent performance when working in time-varying environments but at the cost of increased computational complexity and some stability issues [20]. The above-mentioned approaches work well when the noise is limited to a fixed frequency range. However, if the noise’s frequency is continuously changing, such as those created by motion wander, these methods may not work and their performance with the fixed noise. However, by using a better reference noise signal that is sensitive to motion, one can design an adaptive filter that can automatically cancel the noise for different ECG frequencies. Many researchers, such as [21,22], use accelerometer data frequently associated with mobile commercial ECG sensors to implement those filters.

### 2.2. Proposed Algorithm

**Figure 3:**
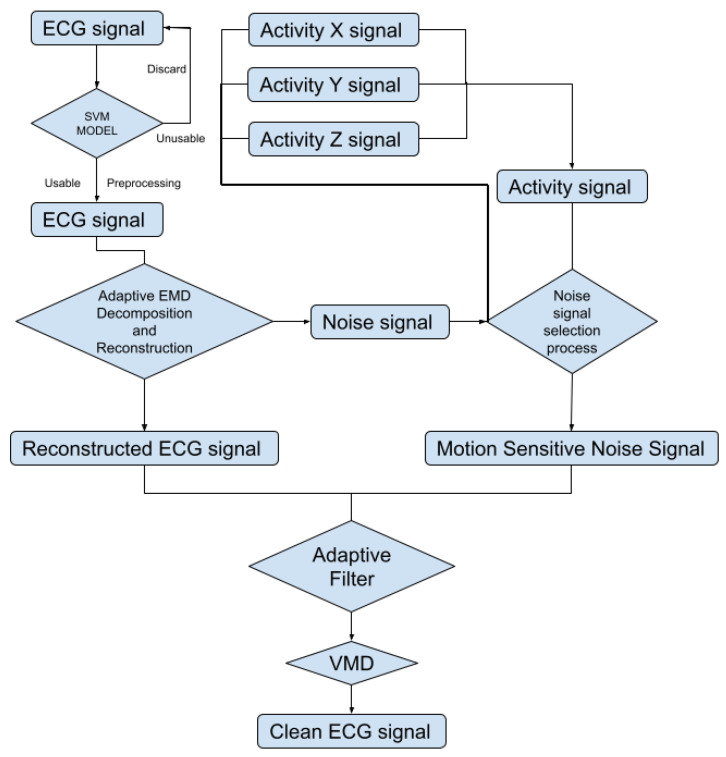
Block diagram of the proposed algorithm.

To automatically reduce the baseline wander and motion artifact, we have developed a new algorithm. The new algorithm consists of two parts: a preprocessing step and a filter step. The preprocessing step contains ECG signal preprocessing (Section 2.2.2) and unusable ECG signal detection (Section 2.2.3). The filter step consists of an adaptive empirical mode decomposition and reconstruction step that could automatically decompose the signal without human input (Section 2.2.4) to reduce motion wander, a motion-sensitive adaptive filter (Section 2.3) that uses a 3-axis accelerometer that automatically selects the best reference noise signal to remove motion artifacts, and a variational mode decomposition and reconstruction method (Section 2.3.1) to remove high-frequency noise. The complete block diagram of the algorithm can be seen in Figure 3.

#### 2.2.1. Data Acquisition

In this research, we used the Astroskin smart shirt [3] and the VIVALNK wearable ECG device [2] for ECG collection. The Astroskin smart shirt can collect a 3-lead ECG at 250 Hz and a 3-axis accelerometer data at 50 Hz. The VIVALNK wearable ECG device can collect a single lead ECG at 128 Hz and a 3-axis accelerometer data at 5 Hz. During the experiment, the ECG signals collected by the VIVALNK ECG device are up-sampled to 250 Hz. Both devices could collect lead II ECG signals and accelerometer signals. The test subject was a healthy male adult of age 32 with no heart problem. Each device was worn by the subject for one hour consecutively. During the one hour, the test subject was free to do his daily activities, including resting, sitting, walking, and cycling. During the resting time, the subject was sitting but not moving his upper body for 5 min. The resting signals were used as the reference signal. The data were collected from the author of this research paper, and therefore, ethical approval was not necessary.

**Figure 4:**
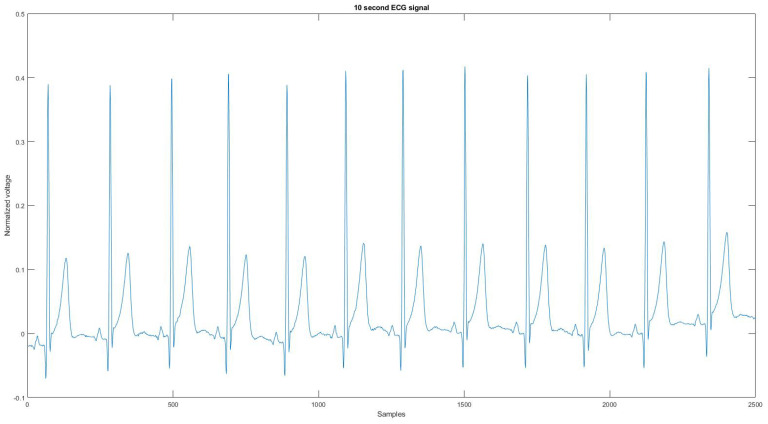
The reference signal from Astroskin smart shirt.

Since the device could not record the noise source or a clean signal for the algorithm to compare, the reference signal is used as the ground truth signal to compare the ECG signal before and after applying the proposed noise removal algorithm. Figure 4 is the reference signal collected from the Astroskin smart shirt, and Figure A21 is the reference signal collected from Vivalnk, which can be seen in the Appendix A. The reference signal in Figure 4 is 10 s long. It contains 12 heartbeats, and its average RR interval is 0.824 s. The reference signal in Figure A21 is 10 s long with 11 heartbeats, and the average RR interval is 0.922 s. During the data collection, the sitting time mainly includes working with computers and eating. Active time includes walking on flat ground, going up and downstairs, and cycling on a stationary bike. During the test for each device, the total sitting time was around 25 min, the total walking time was 15 min, and the total cycling time was 15 min. One reason that the signals are split into 10-s segments is that the proposed algorithm requires enough QRS sequences in the segment for training and classification. Another reason is to allow the algorithm to adapt to changing ECG signals without losing performance.

#### 2.2.2. ECG Signal Preprocessing

Once the data are collected, a preprocessing step is needed. First, the ECG signal is normalized using the z-score normalization method [23], and the 3-axis accelerometer is also normalized using a min–max normalization method. The z-score normalization method normalized the original data *x* based on its mean value μ and standard deviation vσ using the following equation:

(1) $$\hat{x}=\frac{x-\mu }{\sigma }$$

The min–max normalization method normalized the signal into [0,1] using the following equation:

(2) $$\hat{x}=\frac{x-Min(x)}{Max(x)-Min(x)}$$

where $\hat{x}$ is the normalized signal, *x* is the original signal, Min(x) is the minimum value of the signal, and Max(x) is the maximum value of the signal. After the normalization step, the accelerometer signal is over-sampled with the following steps.

- The process first inserts *p* (for example, p=250) zeros to up-sample the signal.
- Then the new signal is filtered by an FIR anti-aliasing filter to match the shape of the original signal. In this part, the Kaiser window method was used to approximate the ideal anti-aliasing filter.
- Finally, *q* (for example, q=50) samples in the up-sampled signal are discarded to obtain the final signal.

The *p* is the target sample frequency, and the *q* is the original sample frequency. Then with the same length for the ECG signal and the accelerometer signal, they are segmented into 10-s segments where the noise removal algorithm is applied.

#### 2.2.3. Detection of Unusable Signal with Support Vector Machine (SVM)

Following the preprocessing step, some sections of the signals were unusable. This may be because, during the ECG collection step, the patient’s movement may be too pronounced, resulting in lost heartbeat information for some signal segments, which is meaningless to continue the noise removal process. Therefore, the purpose of this step is to detect if the signal segments are too noisy. The standard to distinguish a usable signal and an unusable signal is to find if the number of QRS complexes on the ECG signal is the same as the expected number of QRS complexes. If the number of QRS complexes on the ECG signal is less than 60% of the expected number, the segments will be considered unusable. To achieve this objective, a support vector machine (SVM) model was trained using eight features. A support vector machine (SVM) is a computer algorithm that learns a classifier function from labeled data that can then be used to classify new unlabeled data [24]. The SVM algorithm has been proven to be very accurate and efficient for binary classification in many applications, such as [24,25,26]. Another major reason for using SVM is that the SVM does not require a lot of training data. During the training, the kernel function used for the SVM is Gaussian, which the following equation can express:

(3) $$G\left(x_{j},x_{k}\right)=exp(-||x_{j}-x_{k}{\left|\right|}^{2})$$

where $G(x_{j},x_{k})$ is element (j,k) of the Gram matrix, and $x_{j},x_{k}$ are the vectors representing observations *j* and *k* in *x*.

**Figure 5:**
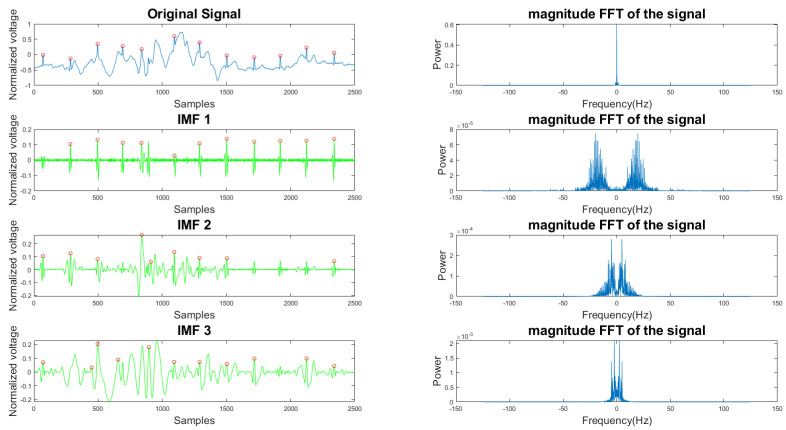
Time and frequency form of a usable ECG signal and its IMFs.

**Figure 6:**
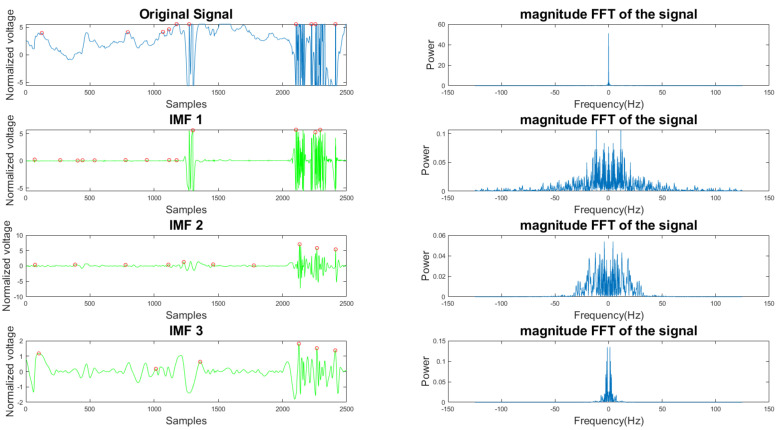
Time and frequency form of an unusable ECG signal and its IMFs.

**Figure 7:**
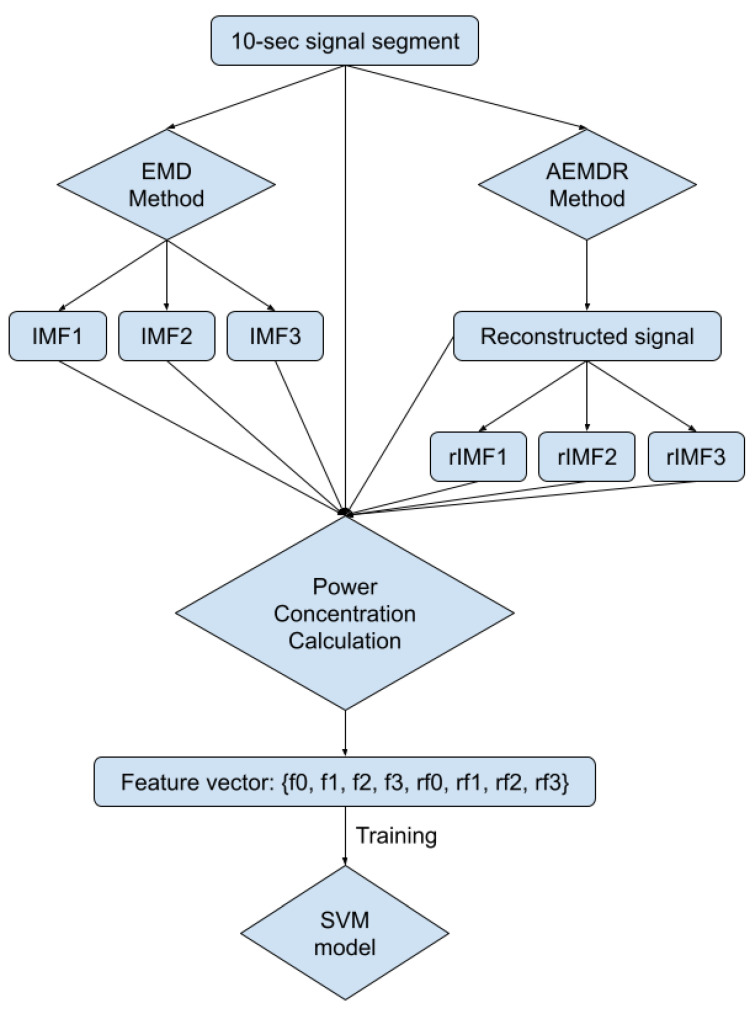
Block diagram of feature extraction and the SVM model training process.

In Figure 5 and Figure 6, the frequency plots show the frequency range for a usable 10-s long ECG signal segment and an unusable 10-s long ECG signal segment. By comparison, one can see that the difference between the two ECG signals is significant. To extract features from the ECG signal, the EMD algorithm, described in Section 2.1, was applied to the ECG signal. During the EMD algorithm, the signal is decomposed into many IMFs, and the first three IMFs contain the most information of the ECG signal. Based on the study of the frequency spectrum, 90% of the frequency components were chosen to represent each signal. These frequency components count from 0 to a frequency *i* and are performed by summation of each frequency power component ($power_{0}$ to $power_{i}$) until they correspond to 90% of the total signal power. The 90% frequency concentration of the original signal (f0) and its corresponding IMF1 (f1), IMF2 (f2), and IMF3 (f3) were calculated. In addition, 90% of the frequency components of the reconstructed signal (rf0) are obtained from adaptive empirical mode decomposition and reconstruction (AEMDR) described in Section 2.2.4, and its corresponding IMF1 (rf1), IMF2 (rf2), and IMF3 (rf3) were calculated. Finally, the following feature vector f0, f1, f2, f3, rf0, rf1, rf2, rf3 is used to represent the ECG signal and to train the SVM model. Figure 7 describes the detail of the feature extraction and training process.

The data for training and testing were collected from both the Astroskin smart shirt and the VIVALNK wearable device. There were 81 unusable ECG segments and 162 usable ECG segments in the training data set. There were 75 useless ECG segments and 75 usable ECG segments in the testing data set. All ECG signal segments were randomly picked from the 1-hour long ECG record. The confusion matrix and the performance are shown in Table 1. The evaluation metrics used are explained here:

- TP: Number of correctly detected usable signals,
- FP: Number of incorrectly detected unusable signals,
- TN: Number of correctly detected usable signals,
- FN: Number of incorrectly detected unusable signal,
- Sensitivity(SEN) = TP/(TP + FN),
- Specificity(SPC) = FN/(FP + TN),
- Accuracy(ACC) = (TP + TN)/(TP + FP + TN + FN).

#### 2.2.4. Adaptive Empirical Mode Decomposition and Reconstruction (AEMDR)

The first step of the algorithm is the adaptive empirical mode decomposition and reconstruction (AEMDR). The purpose of this step is to remove as much low-frequency noise as possible, but in the meantime, the algorithm is trying to preserve the QRS complex as well. The AEMDR is divided into two parts. The first part deals with signal decomposition, and the second part deals with signal reconstruction. For the signal decomposition part, a standard empirical mode decomposition (EMD) algorithm is used. The IMF’s and the lowest frequency component are decomposed using the following rules:

- The number of extremes and zero-crossings must be equal or differ at most by one;
- All local maxima and minima must be symmetric to zero.

After signal decomposition, one wants to reconstruct the high-frequency components as the clean signal and the low-frequency as an estimate of the signal noise. The original EMD algorithm decomposes the signal into *x* IMFs, but *x* is not a fixed number. Thus, to separate clean and noisy IMFs, human intervention is often needed. We are proposing an automatic method to select the clean and noisy IMFs for signal and noise reconstruction to solve this problem.

To achieve our goal, we used the Pan–Tompkins algorithm [27] to detect the QRS complex’s number on the original signal and all the IMFs. The Pan–Tompkins algorithm uses a filter bank that consists of band-pass filters, a differentiator, a squaring filter, and a moving window integrator to reduce the signal noise so that only R wave information is present [7]. The statistical terms are explained as follow:

- The total QRS complex is the number of QRS complexes detected from the original signal with the Pan–Tompkins algorithm.
- The true positive (TP) is the number of QRS complexes detected on the IMFs and also detected on the original signal.
- The false positive (FP) is the number of QRS complexes detected on the IMFs but not on the original signal.

Then we applied the following rules to all IMFs to select the clean ones:

- The IMF is selected when its TP is larger than 50% of the total QRS complex;
- The IMF that satisfies the first condition but has FP that is larger than 50% of the total QRS complex will be applied to the AEMDR method again;
- For the signal reconstruction, if the IMFs meet two conditions: TP is larger than 50% of the total QRS complex; FP is less than 50% of the total QRS complex, then it is considered as a clean IMF; otherwise, it is considered as a noisy IMF.

**Figure 8:**
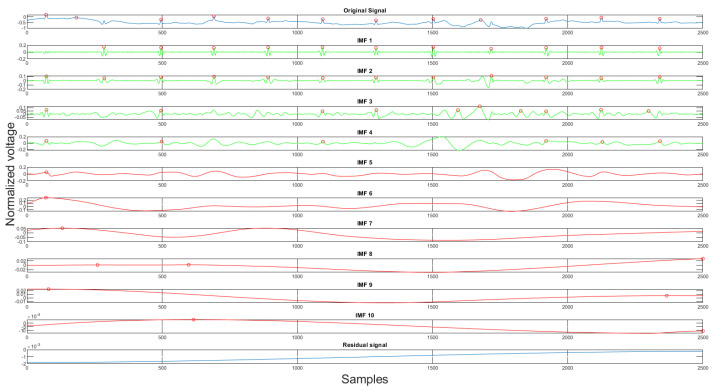
The original signal, clean IMFs, noisy IMFs and residual.

Using the rules discussed earlier, the selected clean IMFs contain more QRS complex information than the noise. Once the clean IMFs and noisy IMFs are selected, the algorithm will proceed with the reconstruction. The decomposition and the IMF selection result can be seen in Figure 8. In the figure, the green IMFs are selected clean signals, the red IMFs are discarded noise signals, and the *x*-axis, *y*-axis are sample numbers and normalized voltage correspondingly. This algorithm will produce a clean signal and a noise signal using the following equations:

(4) $$x\left(n\right)=\sum_{i=1}^{k}c_{i}\left(n\right)$$

(5) $$s\left(n\right)=\sum_{j=1}^{k}c_{j}\left(n\right)+r\left(n\right)$$

where x(n) is the clean signal, s(n) is the noise signal, $c_{i}\left(n\right)$ and $c_{j}\left(n\right)$ the clean IMFs and the noisy IMFs, respectively, m is the number of clean IMFs, and the *k* is the number of noisy IMFs, r(n) is the residual signal. The acquired clean and noise signals are the desired input and reference input processed by the adaptive filter.

#### 2.2.5. Motion-Sensitive Noise Signal Generation

The motion-sensitive noise signal generation step is a preparation step for the following adaptive filter described in Section 2.3. Finding the best reference noise input is always the most challenging task for an adaptive filter application. Removing baseline wander and motion artifacts is harder since it is almost impossible to obtain the noise source from body motion. Some researchers [21,22] explore the possibility of using accelerometer data as the reference noise signal. The accelerometer is standard equipment for many commercial mobile healthcare devices. It can capture the user’s body movement and is used mainly for fall detection. Even though the accelerometer data are related to the motion artifact, it does not perform well when we directly apply it to the adaptive filter. Therefore, we have proposed an algorithm to combine the noise signal extracted from AEMDR analysis with the collected accelerometer signals. By doing so, we could simulate the actual noise on the ECG signal.

The motion noise consists of baseline wander and motion artifacts. The baseline wander noise is a low-frequency noise that is caused by respiration and body motion. The motion artifacts are in the same frequency range as the ECG waves and are caused mainly by the movement of the ECG leads. Our adaptive EMD decomposition and reconstruction algorithm preserves the high-frequency of the original ECG signal and discards the low-frequency noise signal. The discarded noise signal contains the baseline wander information. Then by using the low-frequency noise signal from the AEMDR analysis, the motion artifacts, and the activity signal collected by the accelerometer, one can create a reference signal that the adaptive filter can use.

The next step is to select the activity signal with the highest positive correlation coefficient and the lowest negative correlation coefficient to discriminate unrelated activity signals that interfere with the adaptive filter. The reason for choosing the highest positive correlation coefficient and the lowest negative correlation coefficient is that the two signals are unrelated when the correlation coefficients are closer to zero. We want the reference noise signal to be correlated to the original signal as much as possible. The correlation coefficient is computed using the following equation:

(6) $$\rho \left(A,B\right)=\frac{1}{N-1}\sum_{i=1}^{N}\left(\frac{A_{i}-\mu_{A}}{\sigma_{A}}\right)\left(\frac{B_{i}-\mu_{B}}{\sigma_{B}}\right)$$

where *A* and *B* represent the two signals, and μ and σ are the mean and standard deviation of the corresponding signals, *N* is the total length of the signal.

The activity signals consist of an activity signal on *x*-axis($A_{x}$), activity signal on *y*-axis($A_{y}$) and activity signal on *z*-axis($A_{z}$). The 3-axis combined activity signal $A_{xyz}$ is calculated using the following equation:

(7) $$A_{xyz}=\sqrt{A_{x}^{2}+A_{y}^{2}+A_{z}^{2}}$$

**Figure 9:**
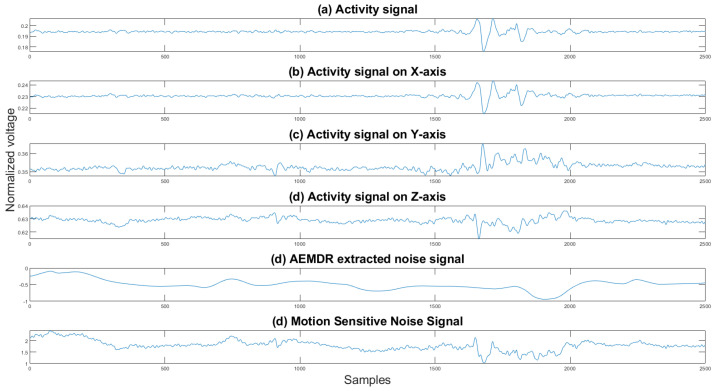
Activity signals, EMD noise signal, and the motion-sensitive reference signal. The *y* axis is the normalized voltage, and the *x* axis is the sample number.

The selected activity signals are combined with the noise signal generated by the EMD algorithm to generate a new noise signal as follows:

(8) $$X\left(n\right)=s\left(n\right)+activity_{xyz/x/y/z}\left(n\right)$$

where X(n) is the final reference noise signal, s(n) is the noise generated by the EMD algorithm, and $activity_{xyz/x/y/z}\left(n\right)$ is the activity signals in the *x*, *y*, and *z* direction, and for all directions by xyz. One can see in Figure 9 an example of the combined noise signal.

### 2.3. Adaptive Filter

**Figure 10:**
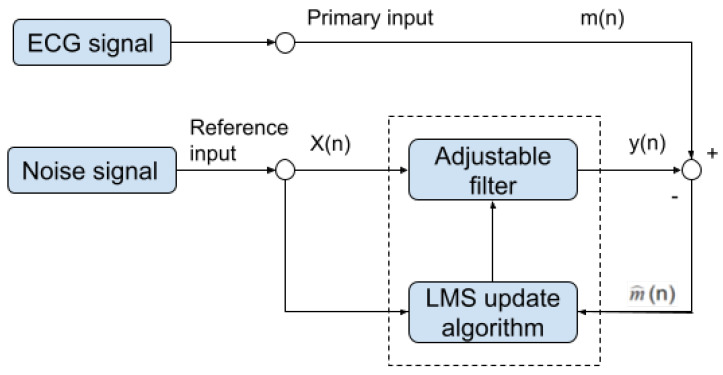
Block diagram of our noise removal adaptive filter.

Once the motion-sensitive noise signal is generated, we can proceed to the next step. This step processes the original signal by an adaptive filter. This step aims to suppress the noise that has a similar frequency as the QRS complex. An adaptive filter is a linear filter where the transfer function is controlled by variable weights that are adjusted according to an optimization algorithm. The closed-loop nature of an adaptive filter uses feedback in the form of an error signal to refine its transfer function, in our case, the combination of the reference noise signal that is sensitive to motion. Generally speaking, the closed-loop adaptive process involves using a cost function, which is a criterion for optimum performance of the filter. One can see in Figure 10 the architecture of our adaptive filter. The measured ECG signal m(n) is composed of the desired signal d(n), which is contaminated by an additive noise $\hat{y}\left(n\right)$ and is defined by:

(9) $$m\left(n\right)=d\left(n\right)+\hat{y}\left(n\right).$$

The main goal of the adaptive filter is to find a noise signal y(n) from a modified reference noise signal X(n) that can minimize the effect of the additive noise $\hat{y}\left(n\right)$, which is defined by:

(10) $$\hat{m}\left(n\right)=d\left(n\right)+\hat{y}\left(n\right)-y\left(n\right).$$

Taking expectation of both sides and realizing that the measured ECG m(n) is uncorrelated with $\hat{y}\left(n\right)$ and y(n) as expressed by:

(11) $$E\left[{\hat{m}}^{2}\right]=E\left[d^{2}\right]+E\left[{\left(\hat{y}-y\right)}^{2}\right]+2E\left[d\left(\hat{y}-y\right)\right]$$

where $E[d\left(\hat{y}-y\right)]=0$. The signal power $E[d^{2}]$ will be unaffected as the filter is adjusted to minimize $E[{\hat{m}}^{2}]$:

(12) $$\min\left[{\hat{m}}^{2}\right]=E\left[d^{2}\right]+\min E\left[{\left(\hat{y}-y\right)}^{2}\right].$$

When the filter is adjusted to minimize the output noise power $E[{\hat{m}}^{2}]$, the output noise power $E[{\left(\hat{y}-y\right)}^{2}]$ is also minimized. Since the output signal remains constant, minimizing the total output power maximizes the output signal-to-noise ratio.

In our implementation, we used the well-known LMS algorithm [28] to optimize the filter parameters for the following reasons. Compared to LMS, NLMS solves the limitation of the LMS with inputs scaling. In our experiment, all signals are scaled, and therefore, the limitations of the LMS filter will not affect the result. We also compared the performance of LMS and RLS algorithms. Both produced a similar result, but because the LMS algorithm complexity 2N+1 is much lower than the recursive least square (RLS) algorithm $4N^{2}$ [29], and the fact that the LMS algorithm converges much faster than the RLS algorithm, we decided to use the LMS algorithm [28]. The LMS algorithm is based on the steepest descent algorithm that adapts the coefficient sample to the sample toward the optimum vector on the performance surface [30]. The LMS algorithm [28] can be described as:

(13) $$W\left(k+1\right)=W\left(k\right)+2\mu X\left(k\right)[m\left(k\right)-X{\left(k\right)}^{T}W\left(k\right)]$$

where W(k) is the weight of the filter of size *M* at iteration *k*, where *M* is the filter order. X(k) is an input vector of size *M* at iteration *k* of the corresponding samples from the reference noise signal X(n), m(k) is the value of the input signal at iteration *k*, the parameter μ is the learning rate (step size). Since $\hat{m}\left(n\right)-d\left(n\right)=\hat{y}\left(n\right)-y\left(n\right)$, this is equivalent to causing the output $\hat{m}\left(n\right)$ to be a best least squares estimate of the signal d(n).

The LMS update algorithm will update the filter weights by minimizing the power of the error signal ${\left(m\left(n\right)-y\left(n\right)\right)}^{2}$. To sum up, using the LMS algorithm, the adjustable filter learns the noise signatures from the original ECG signal. Then, it converts the input noise signal to a new noise signal that can be subtracted from the original ECG signal.

The adaptive filter needs to set two main parameters: learning rate (step size) and the filter order. We have performed a series of experiments to test the best value or range for these two parameters. The learning rate we have tested ranges from 0.005 to 0.05, and the filter order ranges from 1 to 28. Based on the result of our experiments, the learning rate (step size) is set to 0.01, and the filter order is set to be 10.

#### 2.3.1. Variational Mode Decomposition and Reconstruction (VMDR)

**Figure 11:**
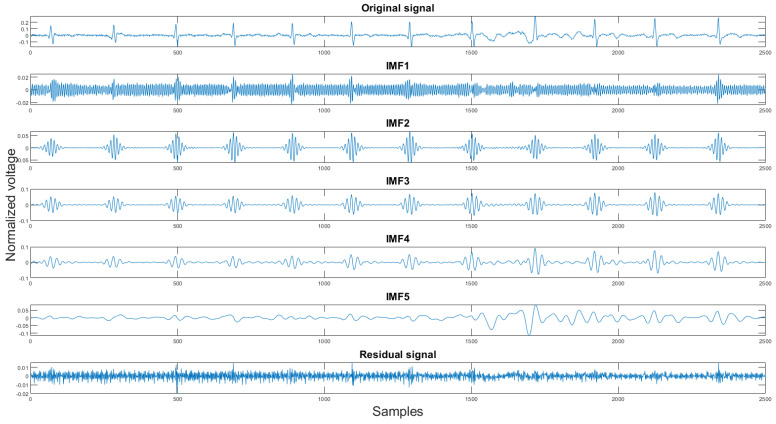
The original signal, IMFs, and residual signal by the VMD method.

Finally, the last step of the proposed algorithm is the variational mode decomposition and reconstruction (VMDR) [16]. The objective of this process is to remove the high-frequency noise on the filtered signal to make the signal more smooth. The variational mode decomposition and reconstruction are divided into two parts: standard variational mode decomposition and the other is selecting clean IMFs for reconstruction. In the decomposition part, the standard VMD method is applied. The variational mode decomposition (VMD) method is similar to the empirical mode decomposition method. It decomposes the signal into *K* different intrinsic mode functions(IMFs). The second part is finding the clean IMFs for reconstruction. As stated in Section 2.1.4, the VMD method produces high-frequency residual signal. This is also proven in our experiment shown in Figure 11. In the figures, the *x*-axis is the sample number, and the *y*-axis is the normalized voltage. Therefore, for the signal reconstruction, the high-frequency residual signal was discarded.

The reason for choosing this method to filter out the high-frequency noise is that, unlike the EMD method, the VMD method is more sensitive towards the high-frequency components [17].

## 3. Validation Metrics and Result Discussions

As described in Section 2.2.1, the data used in this experiment were collected using Astroskin smart shirt and VIVALNK wearable devices. The raw 3-lead ECG signal and its corresponding 3-axis accelerometer signal were acquired for 8 h. However, during the noise removal process, the ECG signals are split into 10-s segments. There were 372 10-s segments obtained from the Astroskin smart shirt and 330 10-s segments acquired from the Vivalnk. After the prediction of the SVM model, 328 segments from the Astroksin and 320 segments from Vivalnk were labeled as usable. There were many unusable ECG segments from Astroskin Smart Shirt because its sensors were not attached to the human body resulting in a loss of contact with the subject’s skin. There was a total of 648 ECG segments of 10-s to be analyzed. However, it is unrealistic to present all of them in this paper. Therefore, we have selected one clean segment and three noisy segments from both devices. The following sections will explain the metrics we used to show how the proposed algorithm improves signal quality. There are three metrics we use to illustrate objectively how the signal was enhanced. These are the histogram of the difference, the correlation coefficients, and the mean square error between the test signal and the reference signal.

To compare the proposed algorithm to the conventional algorithm for motion artifact removal, we have also implemented several noise removal methods based on [21,31]. The reference noise removal algorithms are: a zero-filtering Butterworth high pass filter (IIR) with a cut-off frequency of 0.5 Hz and a filter order of 2, a moving average filter (MA) with window length set as $\frac{samplefrequency}{2*0.5}$; a discrete wavelet transform (DWT) with a Daubechies wavelet of order 8 and 9 levels of decomposition, and the last detail coefficient was discarded; an empirical mode decomposition (EMD) with the previous two intrinsic mode functions discarded; a variational mode composition with *k* equal to the number of IMFs from the EMD method and with the last two IMF discarded as well; and an adaptive filter using only the activity signal (AF).

### 3.1. Visual Result and the Histogram of the Difference between Test Signal and Reference Signals

**Figure 12:**
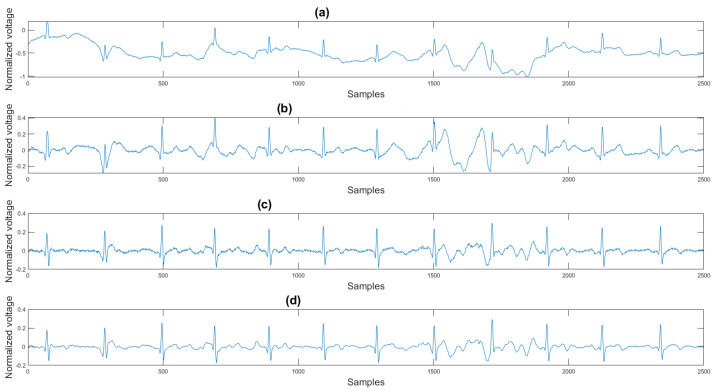
(**a**) Original signal, (**b**) signal after the AEMDR step, (**c**) signal after the adaptive filter step, (**d**) signal after the VMD step.

This section shows the visual result and the histogram of the difference between the test signal and the reference signal. In Figure 12, one can view the result after each step. Figure 12a shows the original signal. The signal contains baseline wander and motion artifacts. Figure 12b is the filter result after the AEMDR step. One can see that the baseline wander was removed, and almost all QRS complexes were in the same baseline. However, the motion artifacts remain in the signal. Then, after applying the adaptive filter step, as in Figure 12c, most of the motion artifacts were compensated for. Finally, the VMD method removes the high-frequency noise components in Figure 12c and produces the final result in Figure 12d.

**Figure 13:**
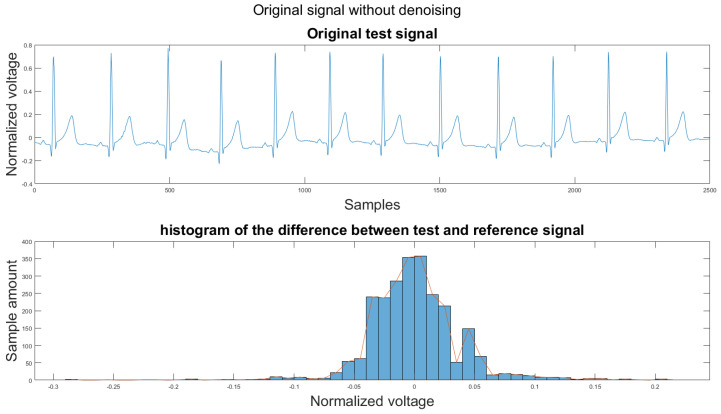
(1) Clean example signal, (2) histogram of the difference.

To set a standard for the histogram of the difference, we have chosen another clean signal taken from the Astroskin smart shirt as an example shown in Figure 13. This figure demonstrates the histogram between two clean signals. Therefore, one can compare the histogram of the denoised signal to Figure 13. If the histogram is similar to the one in Figure 13, then it should be considered as a better denoising result.

One major challenge while calculating the difference is that the heartbeats of the two signal segments were not matched. We annotated all R peaks on both the reference signal and the test signal to solve this problem. Then all RR intervals on the test signal were re-sampled to have the same length as the RR intervals in the reference signal. Finally, the difference between the reference signal and test signal was calculated using the following equation:

(14) D(n)=R(n)−T(n)

where D(n) is the difference signal, R(n) is the reference signal, and T(n) is the test signal.

**Figure 14:**
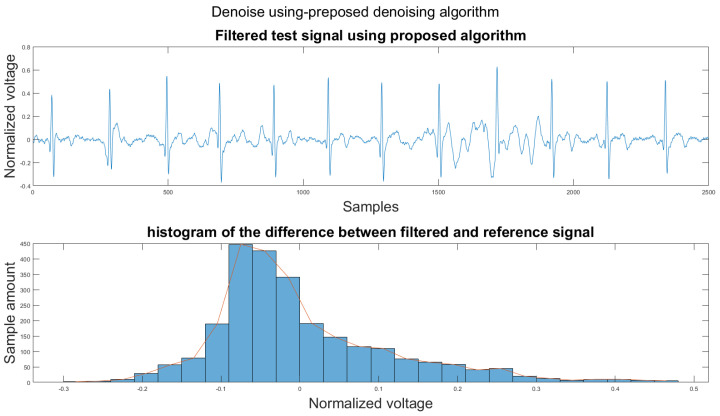
(1) Test signal, (2) histogram of the difference.

**Figure A1:**
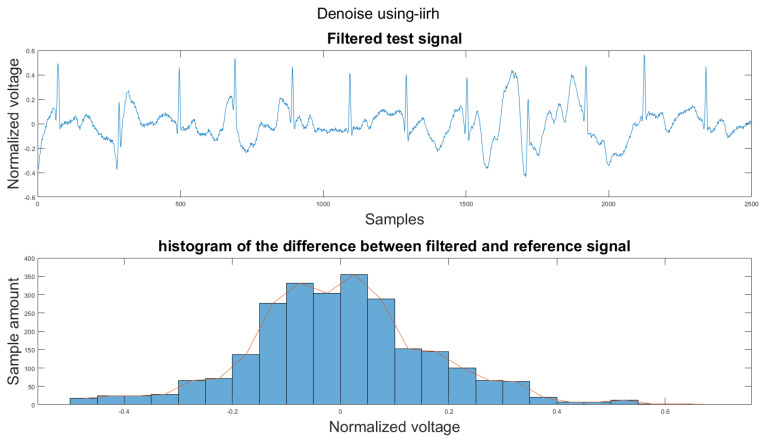
(1) Test signal, (2) histogram of the difference.

**Figure A2:**
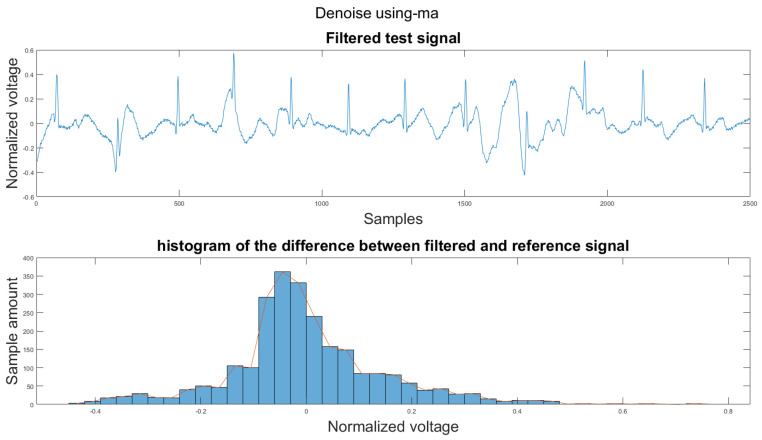
(1) Test signal, (2) histogram of the difference.

**Figure A3:**
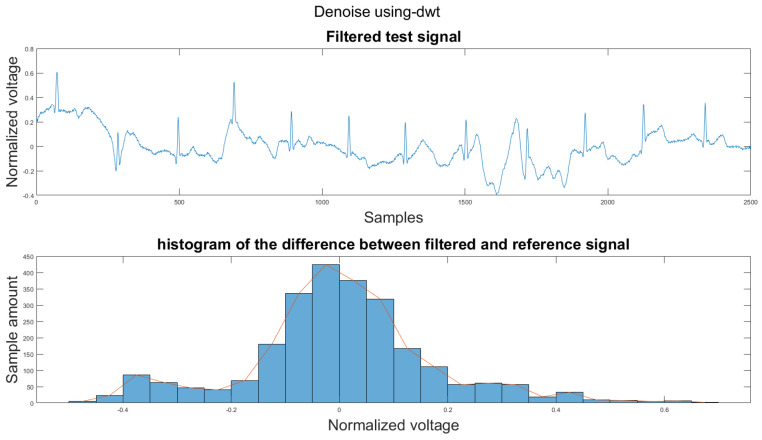
(1) Test signal, (2) histogram of the difference.

**Figure A4:**
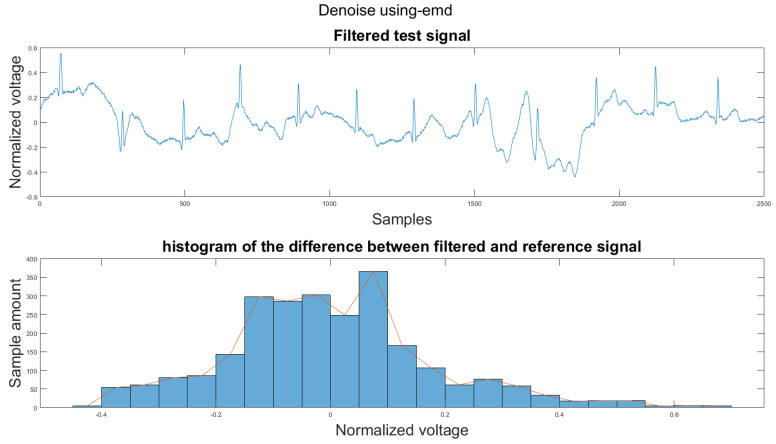
(1) Test signal, (2) histogram of the difference.

**Figure A5:**
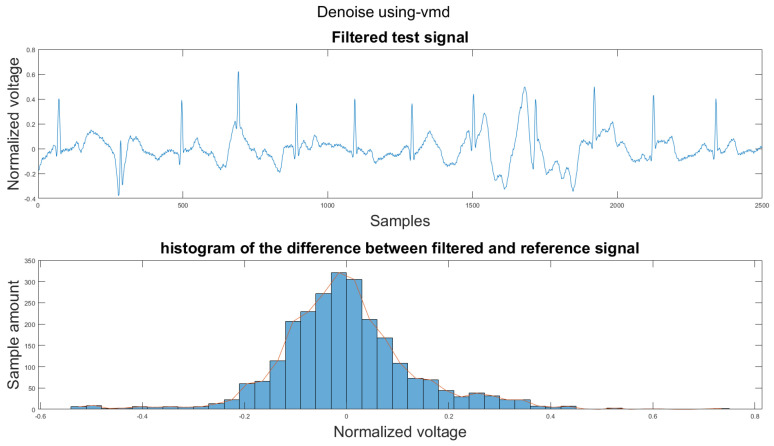
(1) Test signal, (2) histogram of the difference.

**Figure A6:**
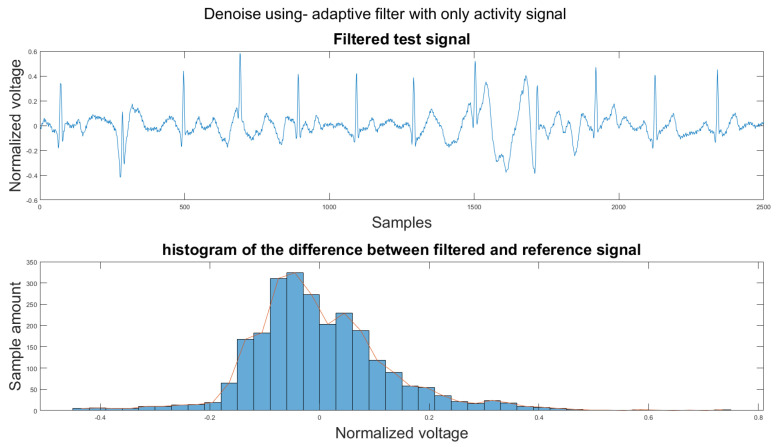
(1) Test signal, (2) histogram of the difference.

**Figure A21:**
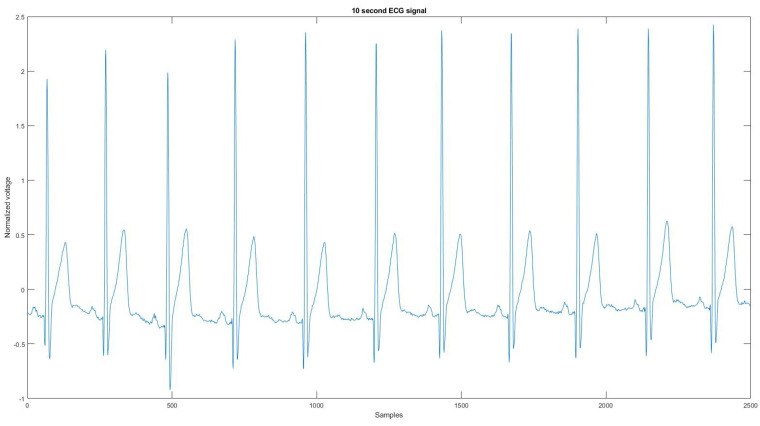
The reference signal from the Vivalnk ECG device.

Figure 14 shows the filter result from the proposed algorithm. To compare our algorithm to other traditional algorithms, Figure A1, Figure A2, Figure A3, Figure A4, Figure A5 and Figure A6 show the filter result by the Butterworth high pass filter, moving average filter, discrete wavelet transform, empirical mode decomposition, variational mode decomposition, and adaptive filter with an accelerometer signal.

### 3.2. Correlation Coefficient

In addition to visual results and the signal-to-noise ratio, we have introduced another metric to validate the algorithms. This metric computes the correlation coefficient between the reference and the test signal. The correlation coefficient ranges from −1 to 1. As the coefficient is closer to 1, the two signals have a more positive relationship. On the other hand, as the coefficient is close to −1, the two signals have a negative relationship. If the coefficient is closer to 0, then the two signals have no relationship. The correlation coefficient is calculated using the following equation:

(15) $$\rho \left(R,T\right)=\frac{1}{N-1}\sum_{i=1}^{N}\frac{R_{i}-\mu_{R}}{\sigma_{R}}\frac{T_{i}-\mu_{T}}{\sigma_{T}}$$

where R,T refer to reference signal and test signal, *N* is the number of samples of the signal (the two signals have the same length), μ is the mean of the signal, and σ is the standard deviation of the signal.

### 3.3. Mean Squared Error

Another metric we have used to compare the algorithms is the mean squared error, which measures the difference between two vectors. If the two vectors are more similar, then the value is close to 0. The mean squared error can be calculated using this equation:

(16) $$\mathrm{MSE}=\frac{1}{n}\sum_{i=1}^{N}{\left(R-T\right)}^{2}$$

where R,T refer to reference signal and test signal, and *N* is the number of samples of the signal.

### 3.4. Results Discussion and Comparison

In Table 2, the first column refers to the figures shown in Section 3.1. The remaining columns show the corresponding mean square error (MSE) and the correlation values between the reference and filtered noisy signals. The bold number indicates the best performance among all noise removal methods. The figures for each example can be found in Appendix A.

According to Section 3.1, Section 3.2 and Section 3.3, both the visual results and the metrics in Table 2 show that the proposed algorithm can remove the baseline wander and suppress motion artifacts to a limit. The histogram of the proposed algorithm always shows a normal Gaussian distribution, which is more similar to the clean signal example in Figure 13.

Therefore, the proposed algorithm can preserve the QRS complex information and perform best when the QRS complex is complete in the ECG signal. The algorithm may not reconstruct the P wave and T wave completely when the noise signal contaminates these two waves, such as in Figure 14. However, if the body motion causes the entire heartbeat to move, this algorithm can put the moved heartbeat back in its location, such as the result examples shown in Section 3.1. According to Table 2, one can see that the signal filtered by the proposed algorithm has a lower MSE and a higher correlation coefficient compared to the algorithms found in the literature. Moreover, based on over 600 segments of 10 s each, the average processing time is 1.5201 s per segment using an average computer programmed in Matlab.

As stated previously, the proposed algorithm clearly shows that one can compensate for baseline wander and motion artifacts on the ECG signal. However, the experimental results are preliminary since only the author’s data were collected and tested. In the future, more subjects need to be tested in a larger clinical trial that would include healthy and cardiac patients. This trial is in preparation at the Mazankowski Alberta Heart Institute but can only be started once the danger related to the COVID-19 pandemic is reduced to a safe level for patients.

## 4. Conclusions

A new method that can automatically remove the baseline wander and reduce the motion artifacts is presented. The algorithm contains five major steps. The first one is the usable ECG signal detection step (Section 2.2.3). This step keeps the ECG signal that can be read and discards unreadable signals using a trained SVM model. The second step is an adaptive empirical mode decomposition and reconstruction algorithm (AEMDR) (Section 2.2.4). It can separate the signal components that contain QRS complex and the components that contain low-frequency noise, such as baseline wander. This step produces an ECG signal and a noise signal. The third is the motion-sensitive signal generation, which combines accelerometer signals and AEMDR extracted noise signals together to form a motion-sensitive signal (Section 2.2.5). This signal is used as the reference signal for the adaptive filter in the fourth step (Section 2.3). This step removes motion artifacts that have a similar frequency range as the QRS complex and produces a cleaned signal. The last step is the variational mode decomposition and reconstruction (VMDR). The method can remove the high-frequency components that are left in the ECG signal to produce a smoother signal (Section 2.3.1). The results in Section 3.1, Section 3.2 and Section 3.3 clearly show improvements in the quality of the test ECG signals compared to various algorithms found in the literature. In addition, the ECG recordings were collected from two different ECG devices: Astroskin smart shirt and the Vivalnk ECG device. Therefore, the proposed noise removal method is robust and can be used for different medical-grade sensors.

## Figures and Tables

**Table 1:** Confusion matrix and performance.

TP	FP	TN	FN	SEN	SPC	ACC
99	2	48	1	99%	96%	98%

**Table 2:** Comparison between different noise removal methods.

Figure	Metric	Original	IIR	MA	DWT	EMD	VMD	AF	Proposed
Figure 14 and Figure A1, Figure A2, Figure A3, Figure A4, Figure A5, Figure A6	CORR	−0.0337	0.2962	0.3169	0.3303	0.4540	0.4395	0.4594	**0.5369**
Figure 14 and Figure A1, Figure A2, Figure A3, Figure A4, Figure A5, Figure A6	MSE	0.0419	0.0326	0.0307	0.1357	0.0090	0.0260	0.0086	**0.0047**
Figure A7, Figure A8, Figure A9, Figure A10, Figure A11, Figure A12 and Figure A13	CORR	0.0651	0.3072	0.3442	0.2507	0.4981	0.4712	0.4988	**0.5902**
Figure A7, Figure A8, Figure A9, Figure A10, Figure A11, Figure A12 and Figure A13	MSE	0.0163	0.0129	0.0123	0.3053	0.0066	0.0092	0.0065	**0.0040**
Figure A14, Figure A15, Figure A16, Figure A17, Figure A18, Figure A19 and Figure A20	CORR	0.1316	0.1846	0.2186	0.1307	0.2719	0.3047	0.3660	**0.4243**
Figure A14, Figure A15, Figure A16, Figure A17, Figure A18, Figure A19 and Figure A20	MSE	0.1091	0.0425	0.0369	0.1000	0.0412	0.0302	0.0092	**0.0054**
Figure A22, Figure A23, Figure A24, Figure A25, Figure A26, Figure A27 and Figure A28	CORR	0.1557	0.3438	0.3181	0.1638	0.1881	0.3031	0.2929	**0.4409**
Figure A22, Figure A23, Figure A24, Figure A25, Figure A26, Figure A27 and Figure A28	MSE	11.1281	1.3549	10.2285	8403	3.0299	0.9603	0.5916	**0.1647**
Figure A29, Figure A30, Figure A31, Figure A 32, Figure A33, Figure A34 and Figure A35	CORR	0.2992	0.4227	0.4393	0.3129	0.4220	0.4759	0.4867	**0.6011**
Figure A29, Figure A30, Figure A31, Figure A 32, Figure A33, Figure A34 and Figure A35	MSE	4.0803	0.7239	0.7073	3.7537	0.9917	0.3016	0.3188	**0.1144**
Figure A36, Figure A37, Figure A38, Figure A39, Figure A40, Figure A41 and Figure A42	CORR	0.2875	0.4844	0.4523	0.051	0.3839	**0.5292**	0.4867	0.5242
Figure A36, Figure A37, Figure A38, Figure A39, Figure A40, Figure A41 and Figure A42	MSE	5.8771	0.3676	0.4371	5.6547	0.3931	0.4822	0.3188	**0.1304**

**Figure A7:**
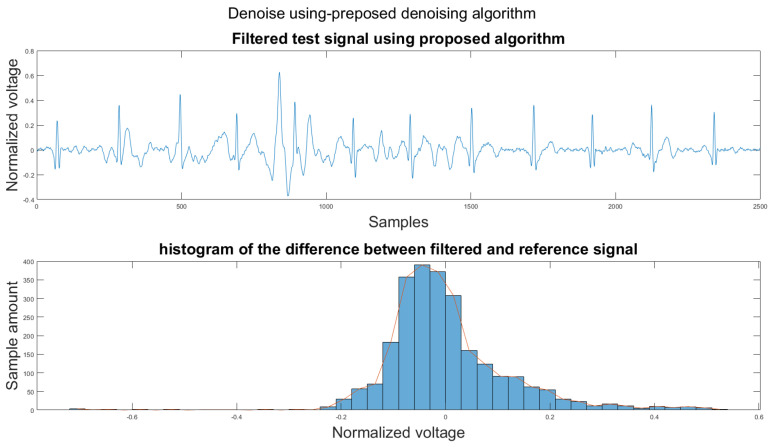
(1) Test signal, (2) histogram of the difference.

**Figure A8:**
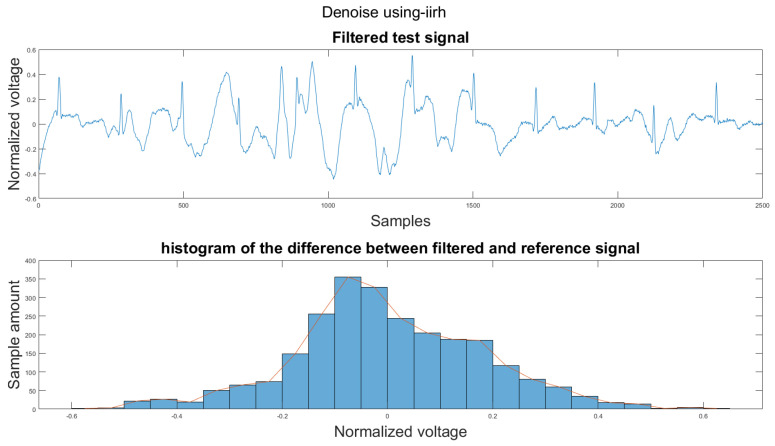
(1) Test signal, (2) histogram of the difference.

**Figure A9:**
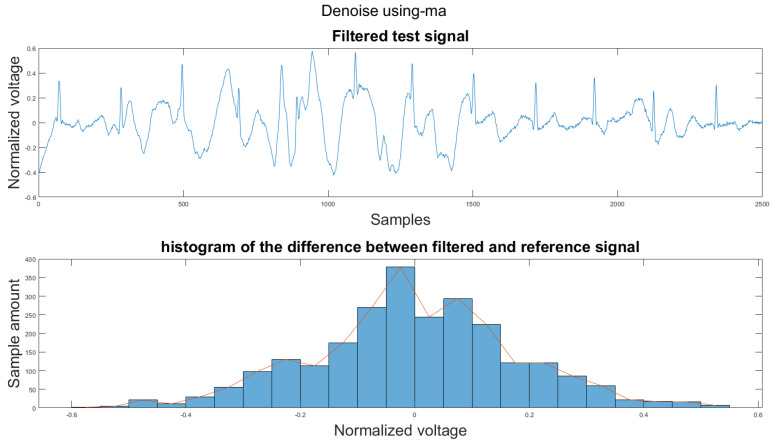
(1) Test signal, (2) histogram of the difference.

**Figure A10:**
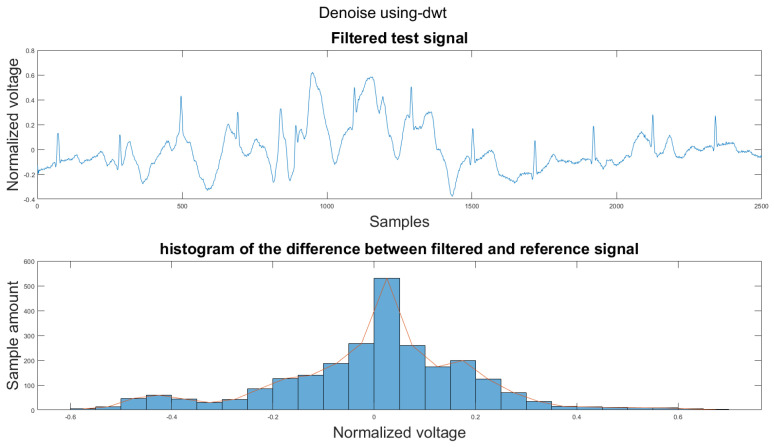
(1) Test signal, (2) histogram of the difference.

**Figure A11:**
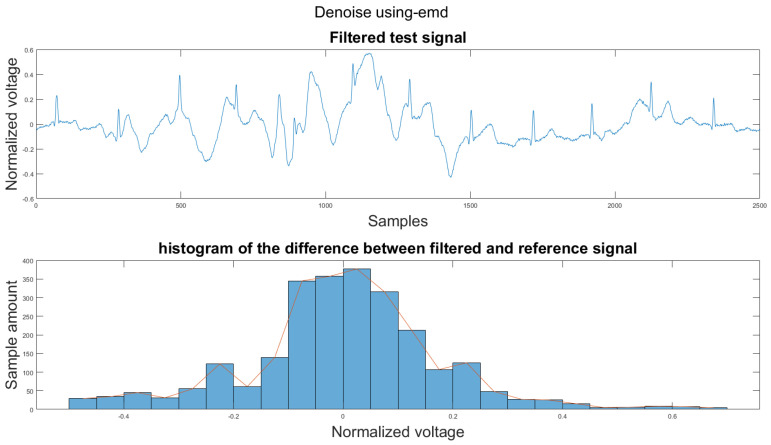
(1) Test signal, (2) histogram of the difference.

**Figure A12:**
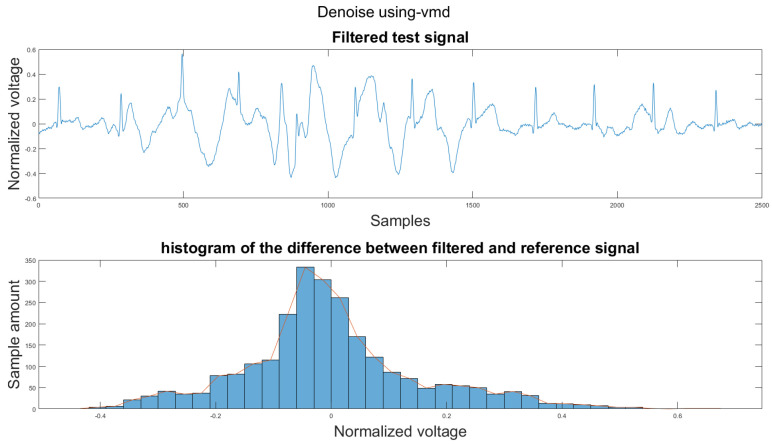
(1) Test signal, (2) histogram of the difference.

**Figure A13:**
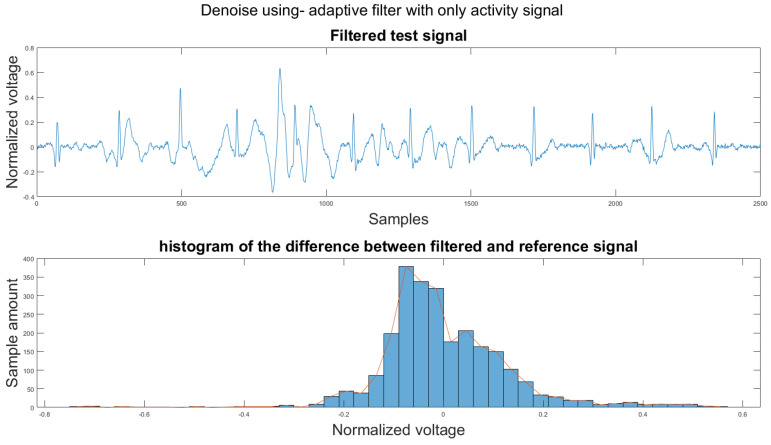
(1) Test signal, (2) histogram of the difference.

**Figure A14:**
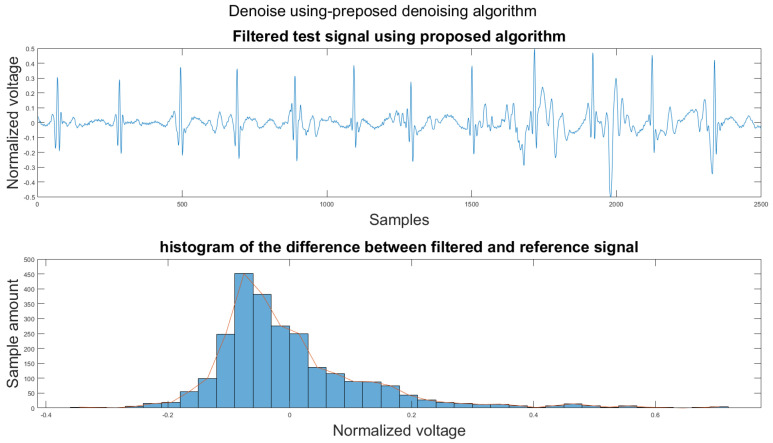
(1) Test signal, (2) histogram of the difference.

**Figure A15:**
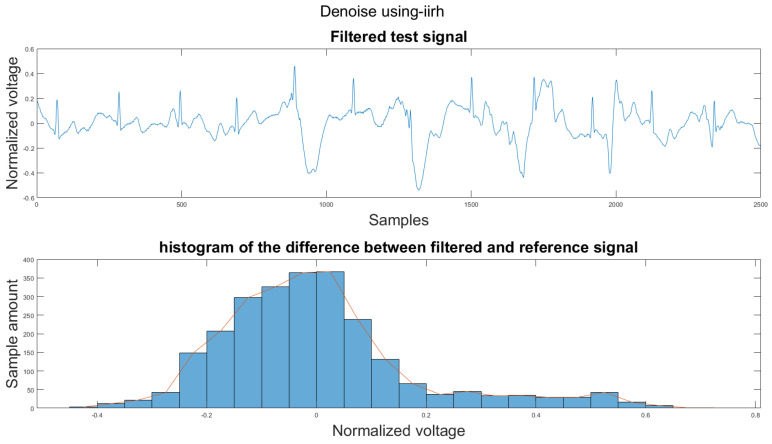
(1) Test signal, (2) histogram of the difference.

**Figure A16:**
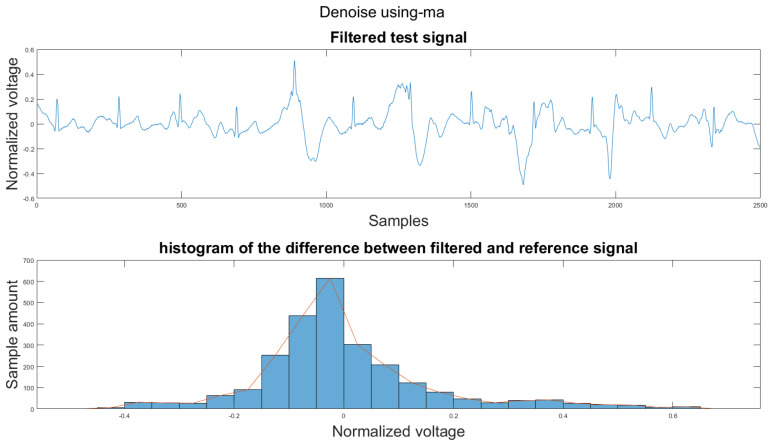
(1) Test signal, (2) histogram of the difference.

**Figure A17:**
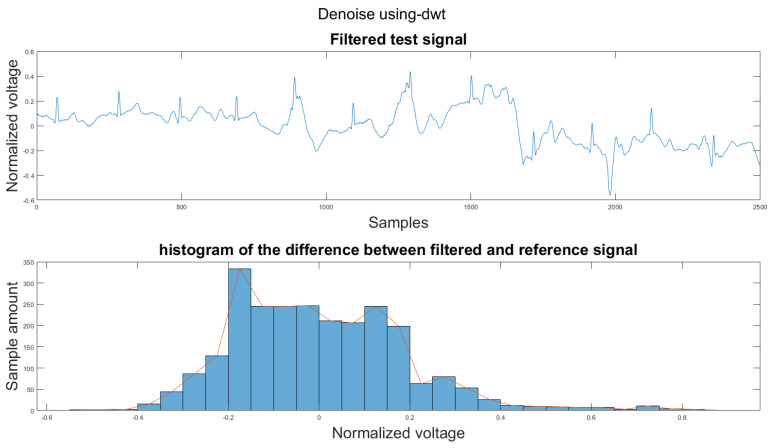
(1) Test signal, (2) histogram of the difference.

**Figure A18:**
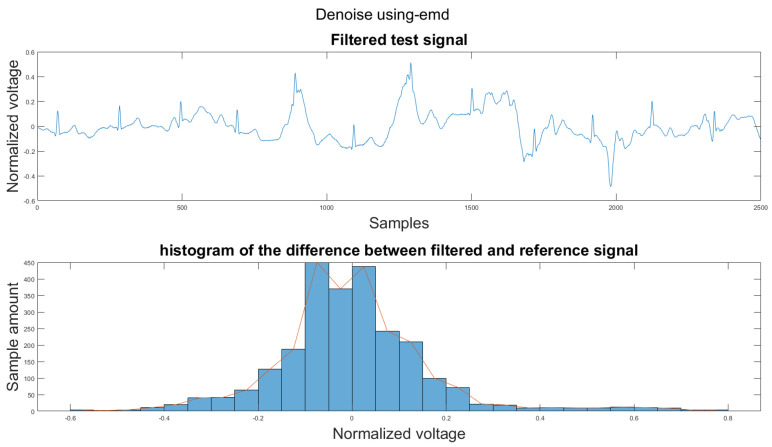
(1) Test signal, (2) histogram of the difference.

**Figure A19:**
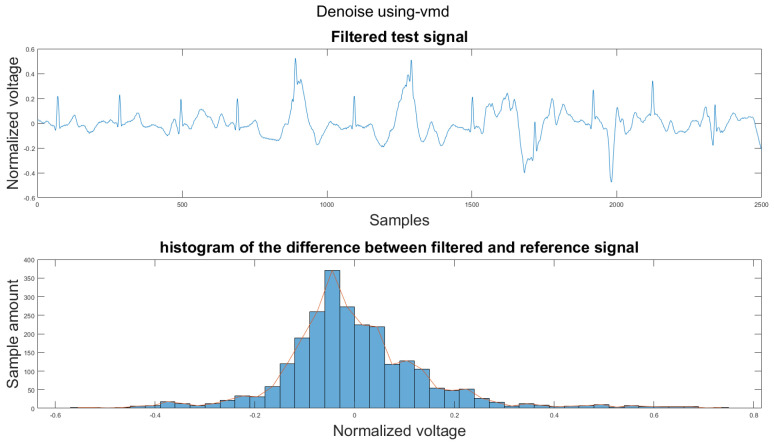
(1) Test signal, (2) histogram of the difference.

**Figure A20:**
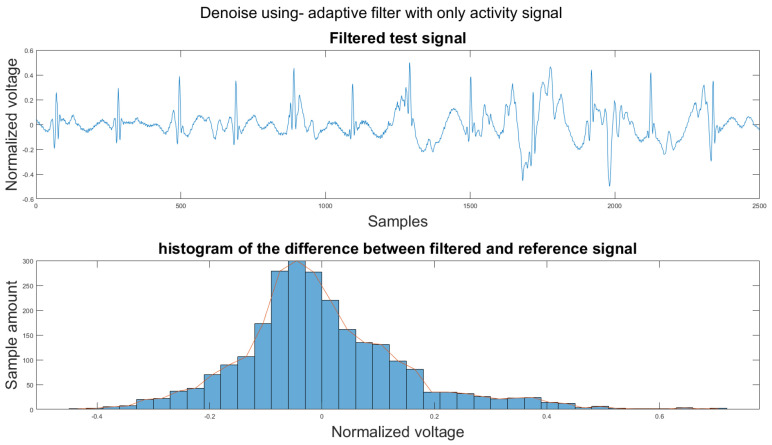
(1) Test signal, (2) histogram of the difference.

**Figure A22:**
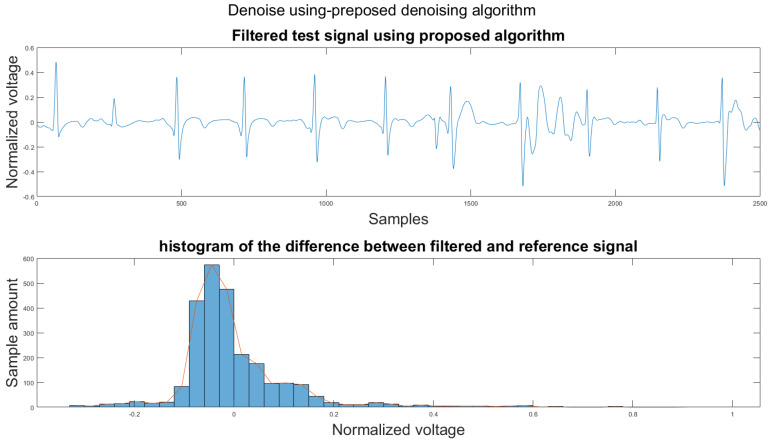
(1) Test signal, (2) histogram of the difference.

**Figure A23:**
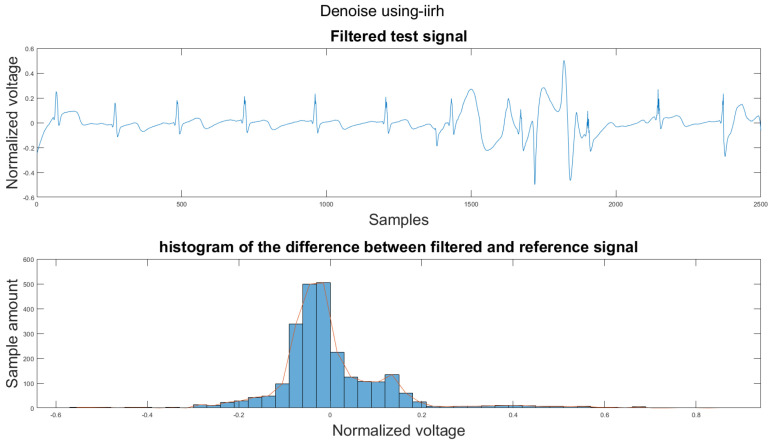
(1) Test signal, (2) histogram of the difference.

**Figure A24:**
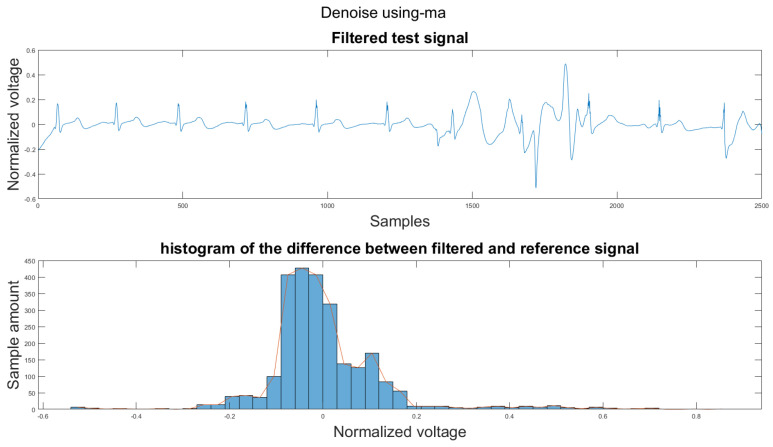
(1) Test signal, (2) histogram of the difference.

**Figure A25:**
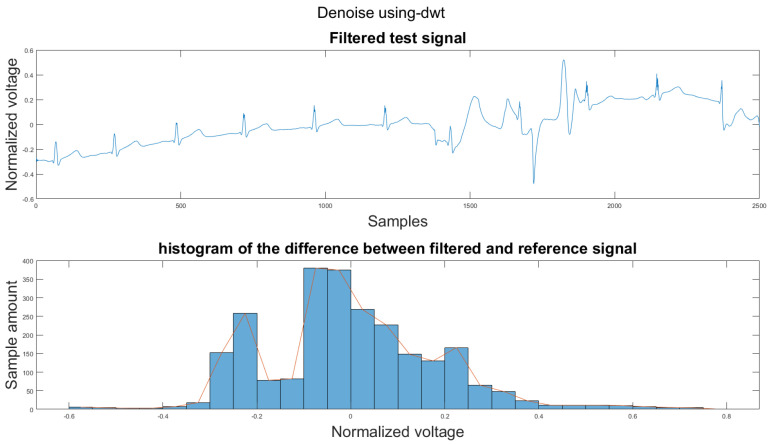
(1) Test signal, (2) histogram of the difference.

**Figure A26:**
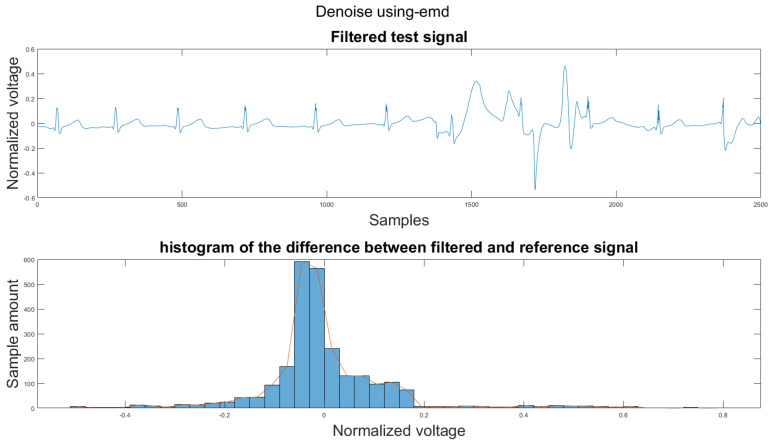
(1) Test signal, (2) histogram of the difference.

**Figure A27:**
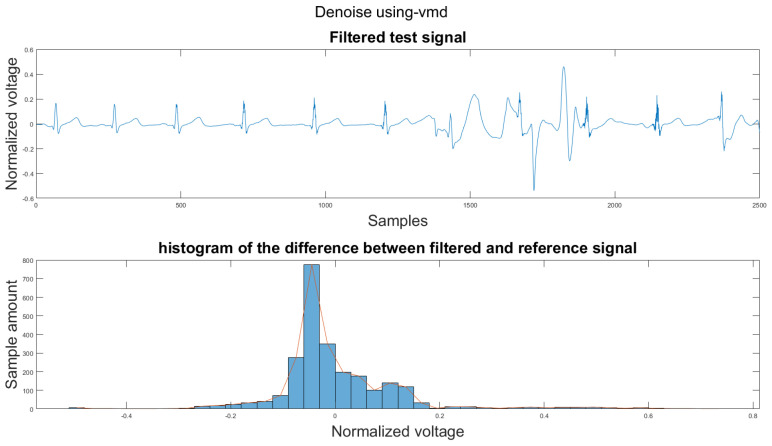
(1) Test signal, (2) histogram of the difference.

**Figure A28:**
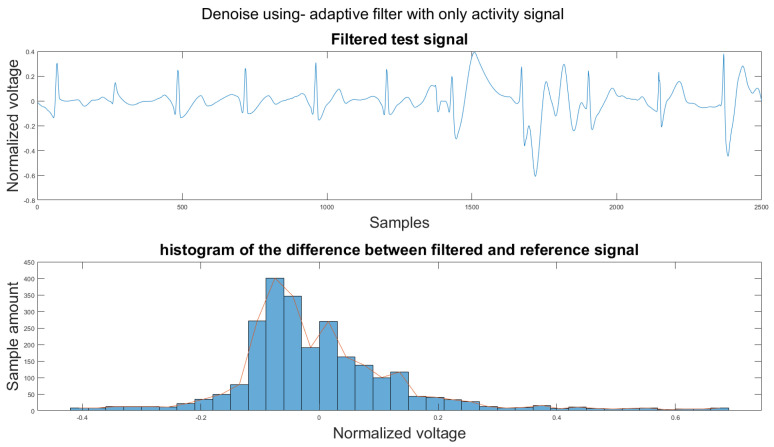
(1) Test signal, (2) histogram of the difference.

**Figure A29:**
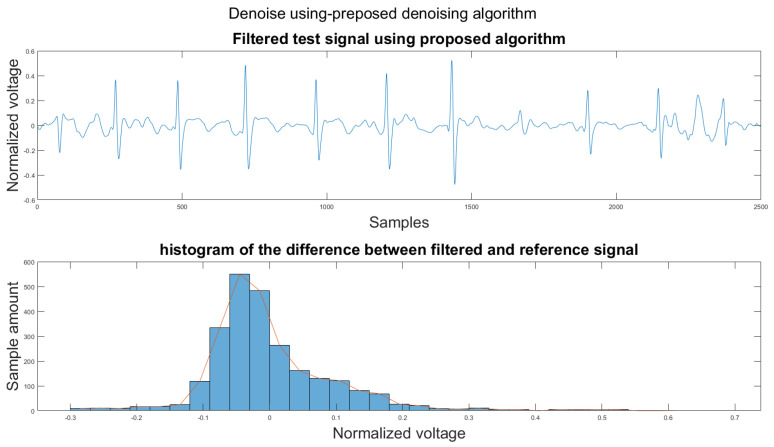
(1) Test signal, (2) histogram of the difference.

**Figure A30:**
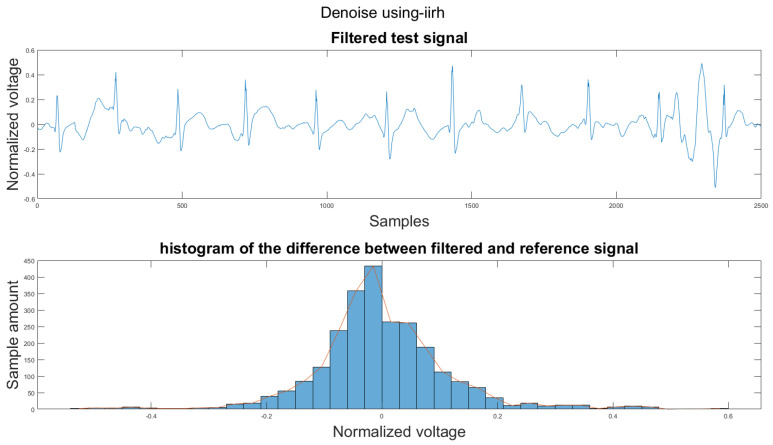
(1) Test signal, (2) histogram of the difference.

**Figure A31:**
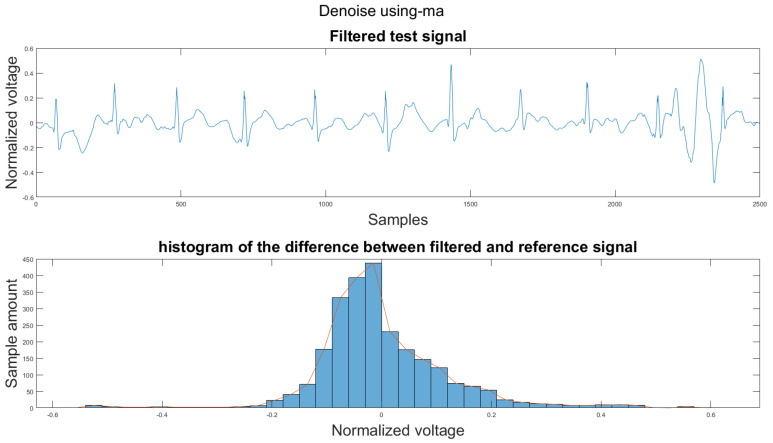
(1) Test signal, (2) histogram of the difference.

**Figure A32:**
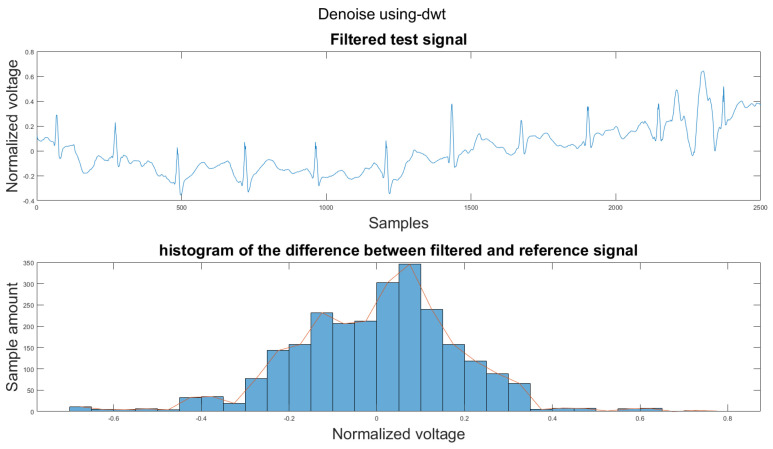
(1) Test signal, (2) histogram of the difference.

**Figure A33:**
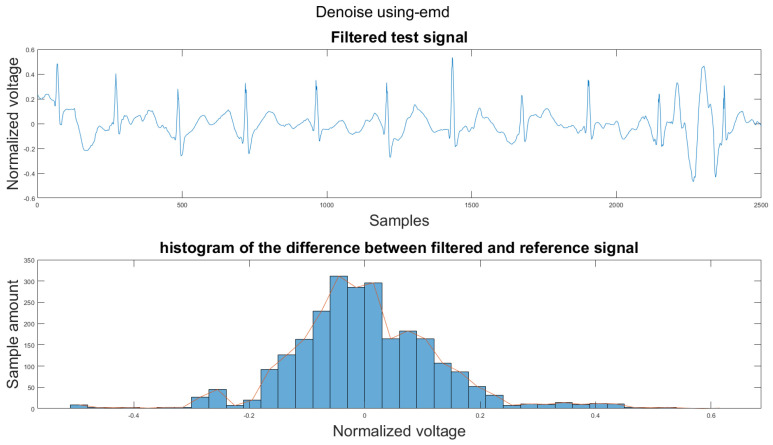
(1) Test signal, (2) histogram of the difference.

**Figure A34:**
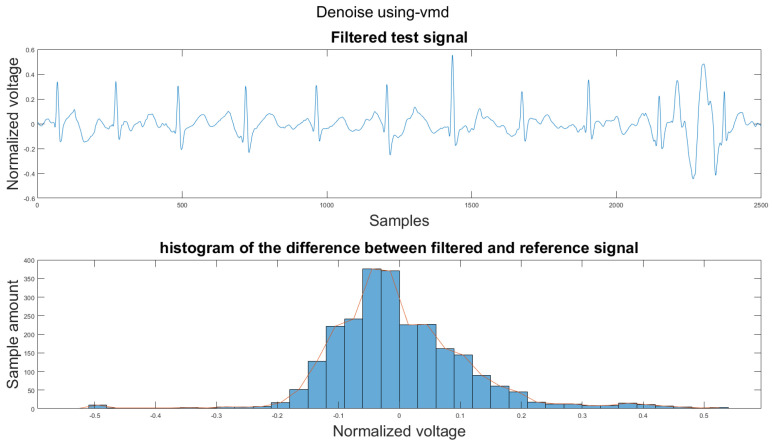
(1) Test signal, (2) histogram of the difference.

**Figure A35:**
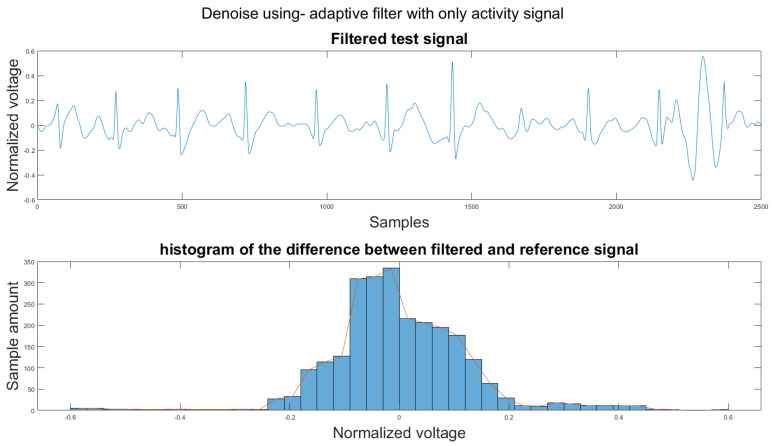
(1) Test signal, (2) histogram of the difference.

**Figure A36:**
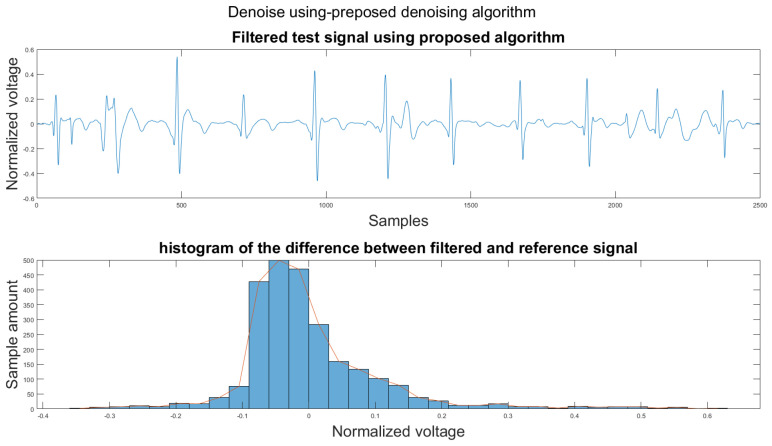
(1) Test signal, (2) histogram of the difference.

**Figure A37:**
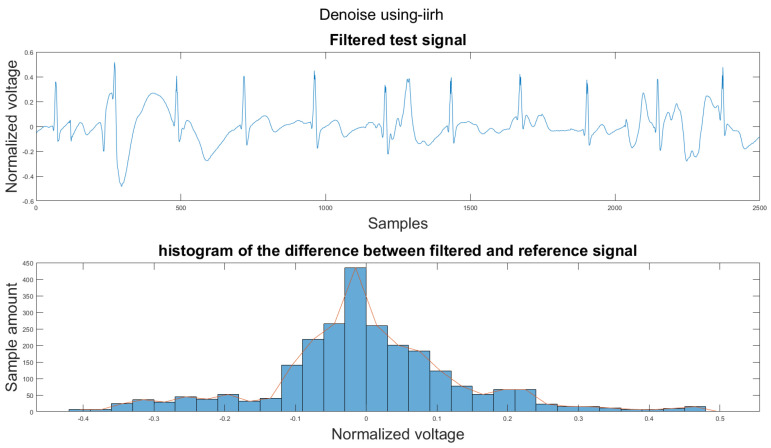
(1) Test signal, (2) histogram of the difference.

**Figure A38:**
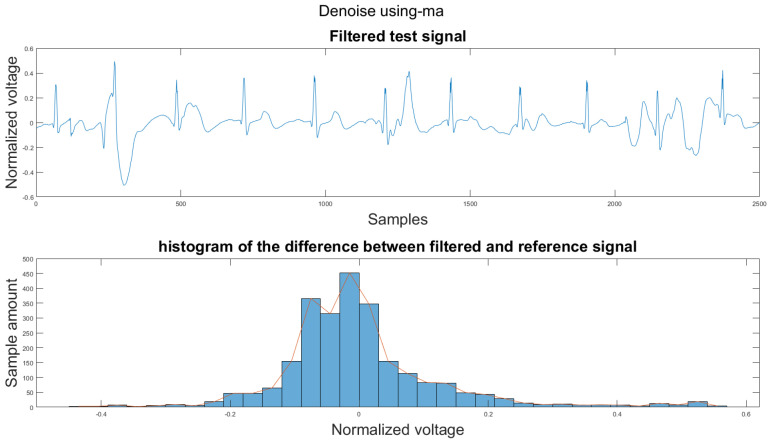
(1) Test signal, (2) histogram of the difference.

**Figure A39:**
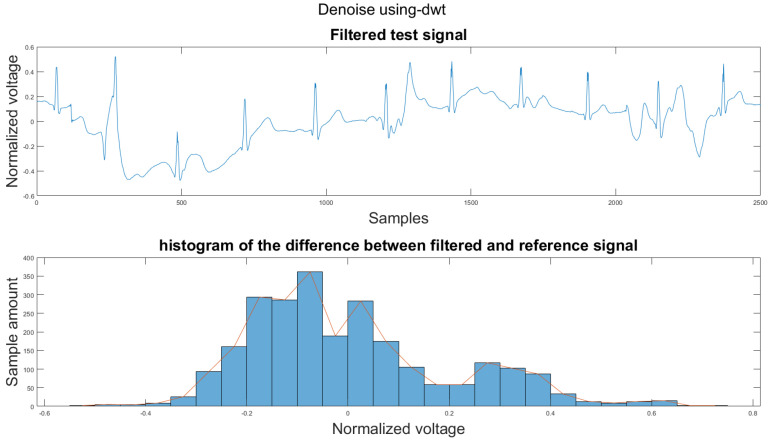
(1) Test signal, (2) histogram of the difference.

**Figure A40:**
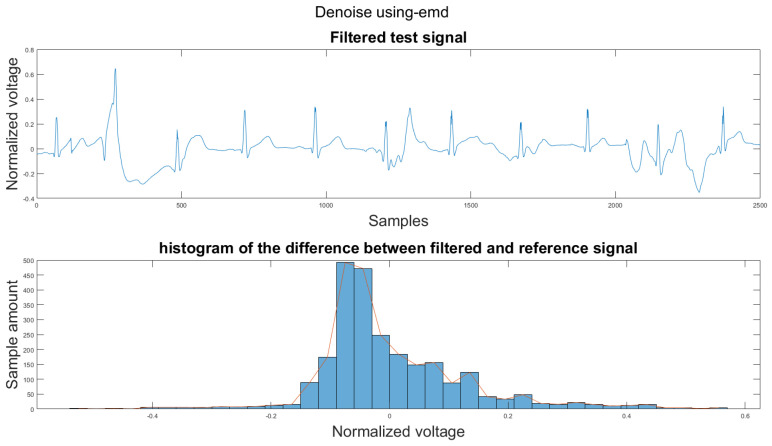
(1) Test signal, (2) histogram of the difference.

**Figure A41:**
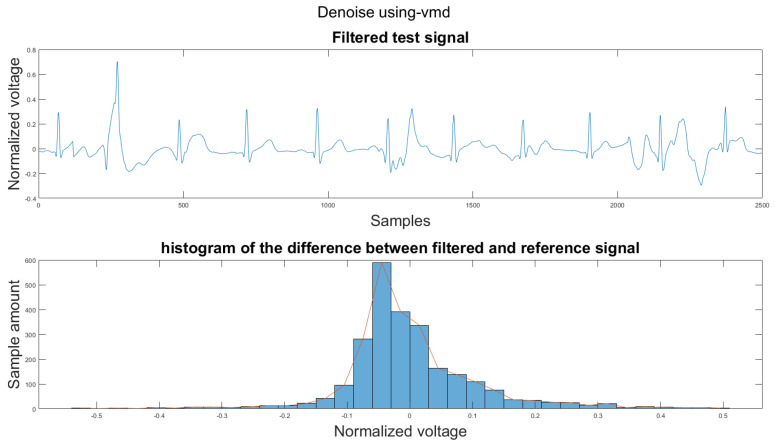
(1) Test signal, (2) histogram of the difference.

**Figure A42:**
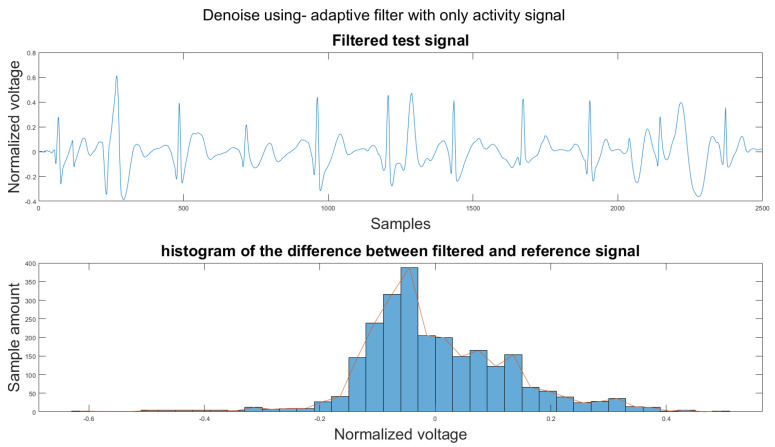
(1) Test signal, (2) histogram of the difference.

## Data Availability

Data used in this research are not provided.

## Appendix A

The appendix contains all the figures that could not be shown in the main pages.
